# Retraction Note: *Tiliroside* as a CAXII inhibitor suppresses liver cancer development and modulates E2Fs/Caspase-3 axis

**DOI:** 10.1038/s41598-025-11770-9

**Published:** 2025-07-29

**Authors:** Rui Han, Hongxing Yang, Lingeng Lu, Lizhu Lin

**Affiliations:** 1 Department of Oncology, The First Affiliated Hospital of Guangzhou University of Chinese Medicine, Guangzhou, 510405 Guangdong People’s Republic of China; 2 Department of Epidemiology and Public Health, Yale University School of Medicine, New Haven, CT 06520 USA; 3 Department of Chronic Disease Epidemiology, School of Medicine, Yale School of Public Health, Yale University, New Haven, CT 06520-8034 USA

Retraction of: *Scientific Reports* 10.1038/s41598-021-88133-7, published online 21 April 2021

The Editors have retracted this Article. After publication irregularities have been identified in the presented figures, specifically:

- In Figure 2A the 0h Hep3B Tiliroside and NC images overlap;
- Images in Figures 3A and B overlap with images in previously published articles [1, 2].

The Authors provided data files to address these concerns; however, the Editors were unable to validate these files as original data. The Editors therefore no longer have confidence in the reliability of the results presented in this Article.

Hongxing Yang and Lingeng Lu have not responded to correspondence from the Editors about this retraction. Rui Han and Lizhu Lin have not explicitly stated if they agree or disagree with this retraction.
